# A case report of Lipoblastoma: Presenting as a swelling in the nape of the neck of 14 years old girl

**DOI:** 10.1016/j.ijscr.2018.09.037

**Published:** 2018-10-04

**Authors:** Shabbar Hussain Changazi, Ghulam Mujtaba Ghuman, Aamir Bashir, Samiullah Bhatti, Mustansar Iqbal, Usman Ismat

**Affiliations:** Services Hospital, Lahore, Pakistan

**Keywords:** Case report, Lipoblastoma, Neck swelling, Adolescence

## Abstract

**Introduction:**

Lipoblastoma usually develops in extremities and trunk during infancy or childhood. Its occurrence in head and neck is rare.

**Case presentation:**

Here we present a case of lipoblastoma in 14 years old female who developed swelling in the nape of her neck. Initially the diagnosis of lymphangioma was made on the basis MRI findings but histopathology after excisional biopsy confirmed it to be lipoblastoma.

**Conclusion:**

Lipoblastoma can rarely present as swelling in the nape of neck in adolescents.

## Highlights

• Lipoblastoma is rare benign tumour of extremities.
• It occurs in infants and early childhood.
• It usually involves subcutaneous tissue.
• In our case lipoblastoma occurred in adolescent girl and presented as a mass in name of neck involving muscles.

## 1 Introduction

The work has been reported in line with the SCARE criteria [1]. Lipoblastoma is a benign tumour of subcutaneous fat. It has two types namely lipoblastomatosis and myxoid lipoblastoma [2,3]. It usually affects infants and young children, with approximately 90% occurring before 3 years of age [4]. It affects males more than females, and is typified by a slowly-growing mass [5]. It typically occurs in the extremities and trunk, and rarely develops in the head and neck and other sites. It is usually presents as solitary nodule with well-defined margins and does not cause any signs and symptoms. On histology it typically contains variably differentiated adipocytes, primitive mesenchymal cells, myxoid matrix, and fibrous trabeculae. It may be confused with myxoid liposarcoma, well differentiated liposarcoma and typical lipoma. The major concern with lipoblastoma in children is to completely excise the tumour without leaving any residual tumour to prevent recurrence [6,7]. Complete excision of tumour is the treatment of choice [8]. Despite the lesions being benign, great difficulty can be encountered in its management because of its tendency to invade the different fascial planes [9]. Lipoblastoma is a tumour with good prognosis despite its potential for local invasion and rapid growth but they do not metastasize [10]. Ultrasound, magnetic resonance imaging, fine needle aspiration, and cytogenetics are important diagnostic tools for this rare tumour [11].

## 2 Case presentation

A 14 years old female presented to OPD with complain of swelling at the nape of neck for the last 9 months. The swelling was small initially and then started increasing gradually in size in last 2 months. The swelling was painless and there was no discharge from swelling. The family history of patient was negative for any such swellings.

On examination, a firm non-tender and normothermic swelling measuring 6 * 8 cm was found in the nape of neck with normal overlying skin. The swelling was non-mobile in both(vertical and horizontal) planes. There were no dilated veins or visible pulsation over swelling and the skin over swelling was pinchable. No bruit was auscultated. The examination for cervical and axillary lymph nodes was unremarkable.

**Fig. 1:**
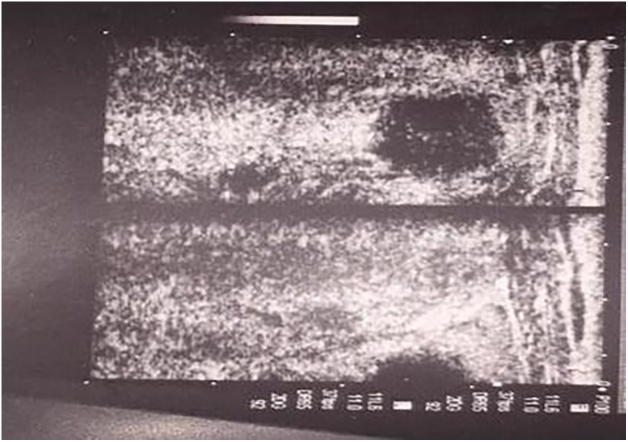
Ultrasound showing cystic lesion at nape of neck.

Ultrasound of neck showed a well capsulated and lobulated mass with cystic areas which contain internal echoes seen at the nape of neck (Fig. 1).

**Fig. 2:**
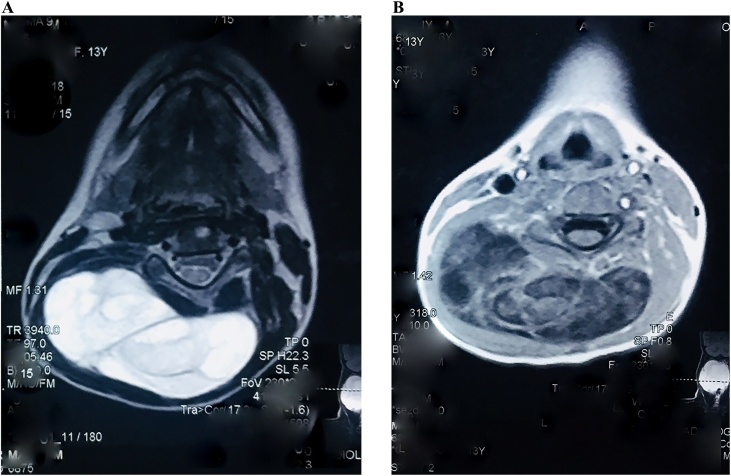
MRI neck T1 and T2 axial images show a multilocular cystic lesion.

MRI neck gives impression of a large lobulated mass lesion approximately measuring 9.2 * 9.1 cm in size at the back of neck with main bulk on right side. Lesion is predominantly cystic with some solid component and appears hyperintense on T2W and FLAIR images, iso to hypointense on T1W images and appearance is suggestive of lymphangioma/cystic hygroma (Fig. 2a, b).

**Fig. 3:**
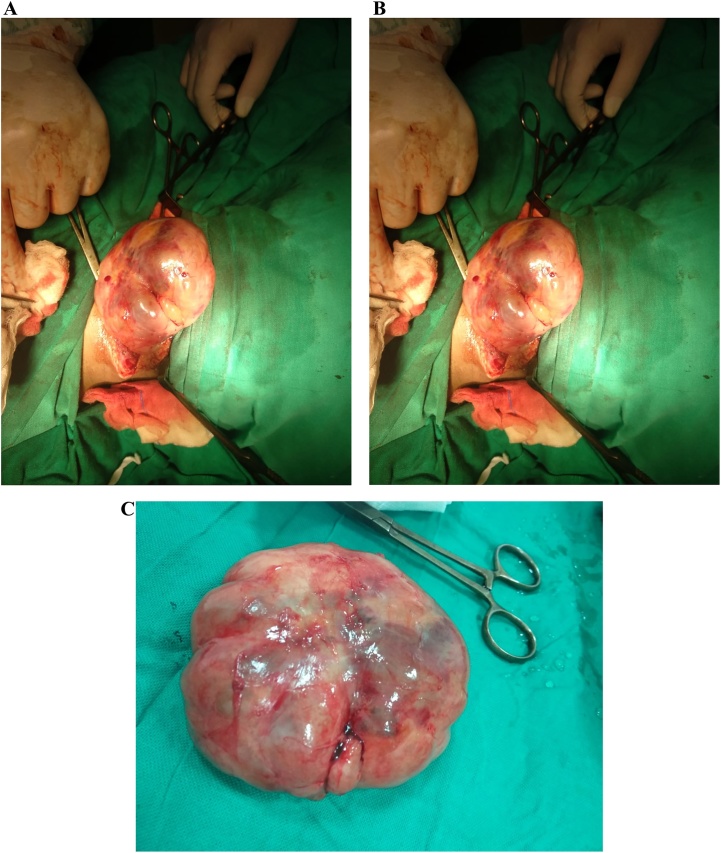
Tumour during surgery and after excision.

Tru-Cut biopsy was taken under aseptic measures from multiple areas and it showed only fibroadipose tissue without any granulomatous or neoplastic process. After detailed discussion, it was decided to go for excisional biopsy of mass under general anesthesia. Skin crease incision was given. Skin and subcutaneous tissue incised. The tumour was located muscle deep against vertebra. So, muscles were incised and tumour delivered to the surface. It was well circumcised mass (Fig. 3a–c). Haemostasis secured and then muscles were closed and then skin closure done.

**Fig. 4:**
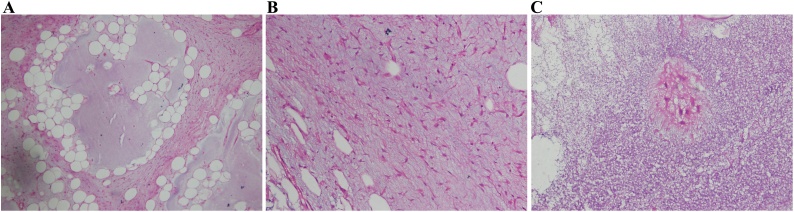
Histopathology of lipoblastoma.

Gross examination of specimen measured a 8.5 * 5 cm mass and histology revealed a circumscribed neoplasm comprised of hypocellular lobules of adipocytes with fibrous septa separating the lobules. Background stroma is myxoid with thin chicken wire vasculature. No atypia, mitoses or necrosis seen. The diagnosis of Lipoblastoma was made (Fig. 4a–c)

Post-operative course and follow up of patient was without any complication.

## 3 Discussion

Lipoblastoma usually occurs during infancy and in young children and affects extremities and trunk. Presentation in adolescents and at the nape of neck is rare. There are only few other cases of occurrence of lipoblastoma in head and neck found during literature review. It is usually subcutaneous but can extend to deeper planes so it is imperative to remove the tumour completely and patient is on follow up to see any recurrence of tumor. In our case lipoblastoma present as swelling in the nape of neck in adolescent girl and also the tumour was muscle deep deep. So this case is unique in its presentation and characteristics.

## 4 Conclusion

Lipoblastoma can rarely present as swelling in the nape of the neck in adolescence and can involve deeper tissue than subcutaneous tissue.

## Conflicts of interest

There are no financial or non-financial competing interest involved with this case report.

## Sources of funding

No funding agency is involved and funds for publication will be contributed by all the authors from their own pockets.

## Ethical approval

It is a case report and ethical approval not required.

## Consent

Written informed consent was obtained from the patient's parent for publication of this case report and accompanying images. A copy of the written consent is available for review by the Editor-in-Chief of this journal on request.

## Author contribution

Dr Shabbar Hussain Changazi and Dr Ghulam Mujtaba Ghuman made substantial contributions to conception and design of data.

Dr Aamir Bashir was involved directly in acquiring data from the patient.

Dr Samiullah Bhatti has been involved in revising it critically for important intellectual content.

Dr Usman Ismat and Dr Mustanasr Iqbal have been involved in drafting the manuscript.

## Registration of research studies

This is a case report and research registration number not required.

## Guarantor

Shabbar Hussain Changazi.

## Provenance and peer review

Not commissioned, externally peer-reviewed.
